# Correction to: Co‑clinical FDG‑PET radiomic signature in predicting response to neoadjuvant chemotherapy in triple‑negative breast cancer

**DOI:** 10.1007/s00259-021-05542-6

**Published:** 2021-09-22

**Authors:** Sudipta Roy, Timothy D. Whitehead, Shunqiang Li, Foluso O. Ademuyiwa, Richard L. Wahl, Farrokh Dehdashti, Kooresh I. Shoghi

**Affiliations:** 1 Department of Radiology, Washington University School of Medicine, St. Louis, MO USA; 2 Department of Medicine, Division of Oncology, Washington University School of Medicine, St. Louis, MO USA; 3 Department of Radiation Oncology, Washington University School of Medicine, St. Louis, MO USA; 4 Department of Biomedical Engineering, Washington University in St. Louis, St. Louis, MO USA

**Correction to: European Journal of Nuclear Medicine and Molecular Imaging**

**https://doi.org/10.1007/s00259-021–05489-8**

The article “Co‑clinical FDG‑PET radiomic signature in predicting response to neoadjuvant chemotherapy in triple‑negative breast cancer”, written by Sudipta Roy, Timothy D. Whitehead, Shunqiang Li, Foluso O. Ademuyiwa, Richard L. Wahl, Farrokh Dehdashti, and Kooresh I. Shoghi, was originally published electronically on the publisher’s internet portal on July 30, 2021 without open access. With the author(s)’ decision to opt for Open Choice, the copyright of the article changed on August 22, 2021 to © The Author(s) 2021 and the article is forthwith distributed under a Creative Commons Attribution 4.0 International License, which permits use, sharing, adaptation, distribution and reproduction in any medium or format, as long as you give appropriate credit to the original author(s) and the source, provide a link to the Creative Commons licence, and indicate if changes were made. The images or other third party material in this article are included in the article's Creative Commons licence, unless indicated otherwise in a credit line to the material. If material is not included in the article's Creative Commons licence and your intended use is not permitted by statutory regulation or exceeds the permitted use, you will need to obtain permission directly from the copyright holder. To view a copy of this licence, visit http://creativecommons.org/licenses/by/4.0/.

The original article has been corrected.

