# Supplementary material for: Co-clinical FDG-PET radiomic signature in predicting response to neoadjuvant chemotherapy in triple-negative breast cancer
Source: Eur J Nucl Med Mol Imaging. 2021 Jul 30;49(2):550–62. doi: 10.1007/s00259-021-05489-8 (PMC8800941; doi:10.1007/s00259-021-05489-8)
Supplement: Supplementary file 1 — Supplementary file1 (DOCX 80 KB) [file 259_2021_5489_MOESM1_ESM.docx]

**Co-clinical FDG-PET Radiomic Signature in Predicting Response to** **Neoadjuvant Chemotherapy in Triple Negative Breast Cancer**

Sudipta Roy^1^, Timothy Whitehead^1^, Shunqiang Li^2^, Foluso O. Ademuyiwa^2^, Richard L. Wahl^1,3^, Farrokh Dehdashti^1^, Kooresh I. Shoghi^1,4^

^1^Department of Radiology, ^2^Department of Medicine, Division of Oncology, and ^3^Department of Radiation Oncology, Washington University School of Medicine, St. Louis, MO, USA; ^4^Department of Biomedical Engineering, Washington University in St. Louis, St. Louis, MO, USA

**Supplemental Table S1:** *Radiomic features.* Light shaded color radiomics were non reproducible features.

| **Type** | **Method** | **Description** | **Sl. NO** | **Features short name** | **Features Full name/description** | LCC value |
| --- | --- | --- | --- | --- | --- | --- |
| First order (33) | Gray-level intensity, histogram, and statistics | Global distribution of intensity values in terms of spread, symmetry, flatness, uniformity and randomness | 1 | SUV25 | Mean of Standardized Uptake Values of top 25% threshold | 0.87 |
|  |  |  | 2 | SUVmean | Mean intensity | 0.91 |
|  |  |  | 3 | SUVmax | Max intensity | 0.94 |
|  |  |  | 4 | SUVP4 | Mean intensity of sphere of radius 1 | 0.92 |
|  |  |  | 5 | SUVP14 | Mean intensity of sphere of radius 2 | 0.90 |
|  |  |  | 6 | SUVP33 | Mean intensity of sphere of radius 3 | 0.87 |
|  |  |  | 7 | SUV_N14 | Mean intensity of top 14 voxels | 0.93 |
|  |  |  | 8 | SUV_N45 | Mean intensity of top 45 voxels | 0.91 |
|  |  |  | 9 | SUV_N90 | Mean intensity of top 90 voxels | 0.89 |
|  |  |  | 10 | Variance | - | 0.89 |
|  |  |  | 11 | Median | - | 0.91 |
|  |  |  | 12 | Minimum | - | 0.86 |
|  |  |  | 13 | 10th percentile | - | 0.89 |
|  |  |  | 14 | 90th percentile | - | 0.92 |
|  |  |  | 15 | Interquartile range | - | 0.92 |
|  |  |  | 16 | Range | - | 0.95 |
|  |  |  | 17 | Mean absolute deviation | - | 0.93 |
|  |  |  | 18 | Robust mean absolute deviation | - | 0.93 |
|  |  |  | 19 | Median absolute deviation | - | 0.93 |
|  |  |  | 20 | CV | Coefficient of Variance | 0.90 |
|  |  |  | 21 | Quartile coefficient of dispersion | - | 0.89 |
|  |  |  | 22 | Energy | - | 0.96 |
|  |  |  | 23 | Root mean square | - | 0.91 |
|  |  |  | 24 | Q Mean | Quartile Mean | 0.70 |
|  |  |  | 25 | Q Variance | Quartile Variance | 0.74 |
|  |  |  | 26 | Q Mean absolute deviation | Quartile Mean absolute deviation | 0.74 |
|  |  |  | 27 | Q Robust mean absolute deviation | Quartile Robust mean absolute deviation | 0.78 |
|  |  |  | 28 | Q Median absolute deviation | Quartile Median absolute deviation | 0.73 |
|  |  |  | 29 | QCV | Quartile CV | 0.76 |
|  |  |  | 30 | Q Energy | Quartile Energy | 0.92 |
|  |  |  | 31 | Q Root mean square | Quartile Root mean square | 0.71 |
|  |  |  | 32 | Entropy | - | 0.80 |
|  |  |  | 33 | Kurtosis-hist | - | 0.91 |
|  |  |  | 34 | Q Median |  | 0.68 |
|  |  |  | 35 | Q Minimum |  | 0.67 |
|  |  |  | 36 | Q 10th percentile |  | 0.65 |
|  |  |  | 37 | Q 90th percentile |  | 0.66 |
|  |  |  | 38 | Q Maximum |  | 0.68 |
|  |  |  | 39 | Q Interquartile range |  | 0.62 |
|  |  |  | 40 | Q Range |  | 0.63 |
|  |  |  | 41 | Q Quartile coefficient of dispersion |  | 0.21 |
|  |  |  | 42 | Global.Variance |  | 0.61 |
|  |  |  | 43 | Global.Skewness |  | 0.67 |
|  |  |  | 44 | Global.Kurtosis |  | 0.62 |
| Second order | Gray-level co-occurrence matrix (GLCM) | Spatial relationship between pixel in a specific direction, highlighting property of uniformity, homogeneity, randomness and linear dependency of images. | 45 | Energy | - | 0.90 |
|  |  |  | 46 | Autocorrelation | - | 0.72 |
|  |  |  | 47 | Contrast | - | 0.97 |
|  |  |  | 48 | Correlation | - | 0.95 |
|  |  |  | 49 | Cluster Prominence | Measure of the skewness and asymmetry | 0.88 |
|  |  |  | 50 | Dissimilarity | - | 0.89 |
|  |  |  | 51 | Entropy | - | 0.85 |
|  |  |  | 52 | Homogeneity1 | - | 0.86 |
|  |  |  | 53 | Homogeneity2 | - | 0.86 |
|  |  |  | 54 | MaximumProbability | Occurrences of the most predominant pair of neighboring intensity values. | 0.87 |
|  |  |  | 55 | SumEntropy | - | 0.80 |
|  |  |  | 56 | DifferenceVariance | - | 0.88 |
|  |  |  | 57 | DifferenceEntropy | - | 0.86 |
|  |  |  | 58 | InfoMesOfCorr1 | Information measures of correlation 1 | 0.83 |
|  |  |  | 59 | InfoMesOfCorr2 | Information measures of correlation 2 | 0.77 |
|  |  |  | 60 | IDN | Inverse Difference Normalized | 0.90 |
|  |  |  | 61 | IDMN | Inverse Difference Moment Normalized | 0.89 |
|  |  |  | 62 | Variance |  | 0.78 |
|  |  |  | 63 | ClusterShade |  | 0.68 |
|  |  |  | 64 | SumOfSqauresVariance |  | 0.67 |
|  |  |  | 65 | SumAverage |  | 0.68 |
|  |  |  | 66 | SumVariance |  | 0.69 |
| Higher order | Gray-level run-length matrix (GLRLM) | Texture in specific direction, where fine texture has more short runs whilst coarse texture presents more long runs with different intensity values. | 67 | SRE | Short Run Emphasis | 0.82 |
|  |  |  | 68 | LRE | Long Run Emphasis | 0.91 |
|  |  |  | 69 | GLN | Gray-Level Non-uniformity | 0.78 |
|  |  |  | 70 | RLN | Run-Length Non-uniformity | 0.87 |
|  |  |  | 71 | RP | Run Percentage | 0.90 |
|  |  |  | 72 | LGRE | Low Gray-Level Run Emphasis | 0.82 |
|  |  |  | 73 | HGRE | High Gray-Level Run Emphasis | 0.77 |
|  |  |  | 74 | SRLGE | Short Run Low Gray-Level Emphasis | 0.81 |
|  |  |  | 75 | LRLGE | Long Run Low Gray-Level Emphasis | 0.82 |
|  |  |  | 76 | LRHGE | Long Run High Gray-Level Emphasis | 0.80 |
|  |  |  | 77 | SRHGE |  | 0.63 |
|  |  |  | 78 | GLV |  | 0.46 |
|  |  |  | 79 | RLV |  | 0.58 |
|  | Gray-level size zone matrix (GLSZM) | Regional intensity variations or the distribution of homogeneity regions. | 80 | LZE | Large Zone Emphasis | 0.93 |
|  |  |  | 81 | GLN | Gray-Level Non-uniformity | 0.89 |
|  |  |  | 82 | ZP | Zone Percentage | 0.87 |
|  |  |  | 83 | LGZE | Low Gray-Level Zone Emphasis | 0.85 |
|  |  |  | 84 | HGZE | High Gray-Level Zone Emphasis | 0.74 |
|  |  |  | 85 | LZLGE | Large Zone Low Gray-Level Emphasis | 0.90 |
|  |  |  | 86 | LZHGE | Large Zone High Gray-Level Emphasis | 0.76 |
|  |  |  | 87 | GLV | Gray-Level Variance | 0.80 |
|  |  |  | 88 | ZSV | Run-Length Variance | 0.90 |
|  |  |  | 89 | SZE |  | 0.50 |
|  |  |  | 90 | ZSN |  | 0.44 |
|  |  |  | 91 | SZLGE |  | 0.61 |
|  |  |  | 92 | SZHGE |  | 0.35 |
|  | Neighborhood gray-tone difference matrix (NGTDM) | Spatial relationship among three or more pixels, closely approaching the human perception of image | 93 | Coarseness | Measure of average difference between the center voxel and its neighborhood | 0.91 |
|  |  |  | 94 | Contrast | Measure of the spatial intensity change on the overall gray level dynamic range | 0.88 |
|  |  |  | 95 | Busyness | Measure of the change from a pixel to its neighbor | 0.91 |
|  |  |  | 96 | Complexity | Rapid changes within gray level intensity | 0.81 |
|  |  |  | 97 | Strength | Measure of the primitiveness in an image | 0.86 |
|  | Gray Level Distance Zone Matrix (GLDZM) | Counts the number of groups of connected voxels with a specific gray level (GL) value and distance to region of interest edge. It contains an extra-level of information compared to GLSZM: it captures the relation between the distance from the edge, zone-size, and GL. | 98 | SDE | Small Distance Emphasis | 0.88 |
|  |  |  | 99 | LDE | Large Distance Emphasis | 0.88 |
|  |  |  | 100 | LGDE | Low Gray Dependence Emphasis | 0.83 |
|  |  |  | 101 | SDLGLE | Small Dependence Low Gray Level Emphasis | 0.83 |
|  |  |  | 102 | SDHGLE | Small Dependence High Gray Level Emphasis | 0.70 |
|  |  |  | 103 | GLNU | Gray Level Non-Uniformity | 0.82 |
|  |  |  | 104 | GLNUN | Gray Level Non-Uniformity Normalized | 0.72 |
|  |  |  | 105 | ZDNU | Size Zone Non-Uniformity | 0.86 |
|  |  |  | 106 | ZDNUN | Size Zone Non-Uniformity Normalized | 0.88 |
|  |  |  | 107 | ZP | Zone Percentage | 0.87 |
|  |  |  | 108 | GLV | Gray Level Variance | 0.82 |
|  |  |  | 109 | ZDV | Zone Distance Variance | 0.87 |
|  |  |  | 110 | ZDE | Zone Distance Emphasis | 0.93 |
|  |  |  | 111 | HGLE |  | 0.68 |
|  |  |  | 112 | LDLGLE |  | 0.56 |
|  |  |  | 113 | LDLGLEn |  | 0.56 |
|  | Neighboring Grey Level Dependence Matrix (NGLDM) | The coarseness of the overall texture. | 114 | LDE | Low Dependence Emphasis | 0.77 |
|  |  |  | 115 | HDE | High Dependence Emphasis | 0.91 |
|  |  |  | 116 | LDLGEGL | Low Dependence Low Gray Emphasis Gray Level | 0.88 |
|  |  |  | 117 | HDLGE | High Dependence Low Gray Emphasis | 0.87 |
|  |  |  | 118 | GLNU | Gray Level Non-Uniformity | 0.92 |
|  |  |  | 119 | DCNU | Dependence counts non-Uniformity | 0.88 |
|  |  |  | 120 | LGLCE |  | 0.10 |
|  |  |  | 121 | HGLCE |  | -0.19 |
|  |  |  | 122 | LDHGE |  | 0.54 |
|  |  |  | 123 | HDHGE |  | 0.49 |
|  |  |  | 124 | GLNUN |  | 0.25 |
|  |  |  | 125 | DCNUN |  | 0.55 |
|  |  |  | 126 | GLV |  | -0.03 |
|  |  |  | 127 | DCV |  | 0.51 |
|  |  |  | 128 | DCE |  | 0.38 |
|  |  |  | 129 | DCEn |  | 0.33 |
|  |  |  | 130 | Volume |  | 0.92 |
|  |  |  | 131 | Metabolic Tumor Volume |  | 0.80 |

**Supplemental Table S2:** *Hierarchical clustering on cross correlation for preclinical and clinical features (dendrogram with distances 3 unit)*

| Sl. NO | Features short name | Preclinical Cluster | Clinical Cluster | Sl. NO | Features short name | Preclinical Cluster | Clinical Cluster |
| --- | --- | --- | --- | --- | --- | --- | --- |
| 1 | SUV25 | 12 | 3 | 66 | SumVariance | 6 | 7 |
| 2 | SUVmean | 5 | 3 | 67 | SRE | 20 | 21 |
| 3 | SUVmax | 12 | 3 | 68 | LRE | 15 | 2 |
| 4 | SUVP4 | 12 | 3 | 69 | GLN | 19 | 21 |
| 5 | SUVP14 | 12 | 3 | 70 | RLN | 20 | 21 |
| 6 | SUVP33 | 12 | 3 | 71 | RP | 20 | 21 |
| 7 | SUV_N14 | 12 | 3 | 72 | LGRE | 19 | 19 |
| 8 | SUV_N45 | 12 | 3 | 73 | HGRE | 8 | 7 |
| 9 | SUV_N90 | 12 | 2 | 74 | SRLGE | 19 | 19 |
| 10 | Variance | 13 | 3 | 75 | LRLGE | 15 | 7 |
| 11 | Median | 5 | 3 | 76 | LRHGE | 10 | 13 |
| 12 | Minimum | 20 | 9 | 77 | SRHGE | 7 | 2 |
| 13 | 10th percentile | 1 | 9 | 78 | GLV | 20 | 21 |
| 14 | 90th percentile | 4 | 3 | 79 | RLV | 20 | 21 |
| 15 | Interquartile range | 13 | 3 | 80 | LZE | 14 | 21 |
| 16 | Range | 13 | 3 | 81 | GLN | 19 | 2 |
| 17 | Mean absolute deviation | 13 | 3 | 82 | ZP | 20 | 20 |
| 18 | Robust mean absolute deviation | 13 | 3 | 83 | LGZE | 20 | 21 |
| 19 | Median absolute deviation | 13 | 3 | 84 | HGZE | 1 | 21 |
| 20 | CV | 15 | 6 | 85 | LZLGE | 14 | 18 |
| 21 | Quartile coefficient of dispersion | 15 | 6 | 86 | LZHGE | 14 | 7 |
| 22 | Energy | 13 | 2 | 87 | GLV | 20 | 18 |
| 23 | Root mean square | 4 | 3 | 88 | ZSV | 20 | 16 |
| 24 | Q Mean | 11 | 5 | 89 | SZE | 1 | 10 |
| 25 | Q Variance | 11 | 5 | 90 | ZSN | 2 | 2 |
| 26 | Q Mean absolute deviation | 11 | 5 | 91 | SZLGE | 20 | 21 |
| 27 | Q Robust mean absolute deviation | 11 | 5 | 92 | SZHGE | 17 | 21 |
| 28 | Q Median absolute deviation | 11 | 5 | 93 | Coarseness | 20 | 21 |
| 29 | QCV | 11 | 8 | 94 | Contrast | 21 | 17 |
| 30 | Q Energy | 14 | 2 | 95 | Busyness | 14 | 2 |
| 31 | Q Root mean square | 11 | 5 | 96 | Complexity | 21 | 16 |
| 32 | Entropy | 13 | 15 | 97 | Strength | 20 | 21 |
| 33 | Kurtosis-hist | 18 | 14 | 98 | SDE | 20 | 21 |
| 34 | Q Median | 15 | 6 | 99 | LDE | 14 | 4 |
| 35 | Q Minimum | 11 | 5 | 100 | LGDE | 20 | 10 |
| 36 | Q 10th percentile | 11 | 12 | 101 | SDLGLE | 20 | 19 |
| 37 | Q 90th percentile | 5 | 1 | 102 | SDHGLE | 17 | 10 |
| 38 | Q Maximum | 11 | 5 | 103 | GLNU | 14 | 21 |
| 39 | Q Interquartile range | 11 | 5 | 104 | GLNUN | 3 | 10 |
| 40 | Q Range | 11 | 5 | 105 | ZDNU | 14 | 4 |
| 41 | Q Quartile coefficient of dispersion | 11 | 5 | 106 | ZDNUN | 20 | 2 |
| 42 | Global.Variance | 11 | 8 | 107 | ZP | 20 | 6 |
| 43 | Global.Skewness | 16 | 15 | 108 | GLV | 20 | 1 |
| 44 | Global.Kurtosis | 18 | 14 | 109 | ZDV | 14 | 21 |
| 45 | Energy | 6 | 1 | 110 | ZDE | 14 | 21 |
| 46 | Autocorrelation | 20 | 21 | 111 | HGLE | 17 | 13 |
| 47 | Contrast | 20 | 21 | 112 | LDLGLE | 9 | 4 |
| 48 | Correlation | 10 | 1 | 113 | LDLGLEn | 14 | 8 |
| 49 | Cluster Prominence | 21 | 20 | 114 | LDE | 20 | 21 |
| 50 | Dissimilarity | 8 | 17 | 115 | HDE | 15 | 2 |
| 51 | Entropy | 15 | 21 | 116 | LDLGEGL | 20 | 11 |
| 52 | Homogeneity1 | 15 | 2 | 117 | HDLGE | 14 | 12 |
| 53 | Homogeneity2 | 14 | 1 | 118 | GLNU | 14 | 21 |
| 54 | MaximumProbability | 8 | 1 | 119 | DCNU | 14 | 21 |
| 55 | SumEntropy | 21 | 1 | 120 | LGLCE | 2 | 2 |
| 56 | DifferenceVariance | 21 | 5 | 121 | HGLCE | 18 | 2 |
| 57 | DifferenceEntropy | 8 | 5 | 122 | LDHGE | 1 | 2 |
| 58 | InfoMesOfCorr1 | 3 | 5 | 123 | HDHGE | 13 | 13 |
| 59 | InfoMesOfCorr2 | 15 | 1 | 124 | GLNUN | 20 | 2 |
| 60 | IDN | 15 | 16 | 125 | DCNUN | 16 | 21 |
| 61 | IDMN | 10 | 16 | 126 | GLV | 18 | 12 |
| 62 | Variance | 16 | 2 | 127 | DCV | 9 | 2 |
| 63 | ClusterShade | 1 | 21 | 128 | DCE | 7 | 1 |
| 64 | SumOfSqauresVariance | 6 | 11 | 129 | DCEn | 2 | 21 |
| 65 | SumAverage | 6 | 11 |  |  |  |  |

**Supplemental Table S3:** AIC and BIC stats for volume dependent radiomic features

| **Features name** | **rho(ρ)** | **Stats** | **Linear** | **Exponential** | **Log** | **Power** | **Polynomial** |
| --- | --- | --- | --- | --- | --- | --- | --- |
| Q Energy | 0.98 | AIC | **459.3** | 577.0 | 531.2 | 461.9 | 460.5 |
|  |  | BIC | **477.6** | 595.3 | 549.6 | 480.3 | 485.0 |
| GLCM.Correlation | -0.98 | AIC | -1281.3 | -1299.1 | -1193.8 | **-1304.3** | -1229.8 |
|  |  | BIC | -1262.9 | -1280.8 | -1175.4 | **-1285.9** | -1205.3 |
| GLCM.Contrast | -0.97 | AIC | -642.2 | -648.3 | -681.5 | **-686.7** | -658.3 |
|  |  | BIC | -623.8 | -629.9 | -663.1 | **-668.3** | -633.8 |
| GLSZM.GLV | -0.98 | AIC | -945.1 | -954.8 | -993.0 | **-905.8** | -811.3 |
|  |  | BIC | -926.7 | -936.4 | -974.7 | **-887.5** | -786.8 |
| GLSZM.ZSV | -0.96 | AIC | -822.1 | -832.7 | -868.6 | **-925.9** | -830.2 |
|  |  | BIC | -803.7 | -814.3 | -850.2 | **-907.5** | -805.7 |
| NGTDM.Busyness | 0.97 | AIC | **-68.5** | -16.4 | -21.3 | -60.0 | -60.8 |
|  |  | BIC | **-86.9** | -34.8 | -39.6 | -78.4 | -85.3 |
| NGTDM.Strength | -0.97 | AIC | -53.6 | -63.2 | -118.8 | **-156.6** | -86.1 |
|  |  | BIC | -35.3 | -44.8 | -100.5 | **-138.2** | -61.6 |
| GLDZM.GLNU | 0.97 | AIC | 129.7 | 218.9 | 171.9 | **121.5** | 137.7 |
|  |  | BIC | 148.0 | 237.3 | 190.3 | **139.9** | 162.2 |
| NGLDM.GLNU | 0.99 | AIC | **340.7** | 772.6 | 728.3 | 477.2 | 334.9 |
|  |  | BIC | **359.1** | 791.0 | 746.7 | 495.6 | 359.4 |
| NGLDM.DCNU | 0.99 | AIC | **772.3** | 993.0 | 958.3 | 1079.6 | 752.6 |
|  |  | BIC | **790.7** | 1011.3 | 976.7 | 1098.0 | 777.1 |

* Selected model is highlighted in bold

**Supplemental Table S4:** *Rank importance (Using ReliefF) of radiomic features based on treatment response prediction ( 94 reproducible features, tumor volume and metabolic tumor volume)*

| **Rank** | **Features name for baseline (BL)** | **Features name for difference(4D-BL)** |
| --- | --- | --- |
| 1 | 'GLDZM_GLNUN' | QRootMeanSquare' |
| 2 | 'GLDZM_SDLGLE' | 'QMean' |
| 3 | 'GLDZM_LGDE' | 'QMedianAbsoluteDeviation' |
| 4 | 'QRobustMeanAbsoluteDeviation' | 'QMeanAbsoluteDeviation' |
| 5 | 'QMedianAbsoluteDeviation' | 'QRobustMeanAbsoluteDeviation' |
| 6 | 'QMeanAbsoluteDeviation' | 'Entropy' |
| 7 | 'QMean' | 'QCV' |
| 8 | 'Entropy' | 'QVariance' |
| 9 | 'QRootMeanSquare' | 'SUV_N90' |
| 10 | 'QVariance' | 'SUV25' |
| 11 | 'GLSZM_GLN' | 'SUVP4' |
| 12 | 'GLSZM_GLV' | 'SUV_N45' |
| 13 | 'GLSZM_ZSV' | 'SUVmax' |
| 14 | 'QCV' | 'SUV_N14' |
| 15 | 'GLCM_Contrast' | 'GLCM_Correlation' |
| 16 | 'NGLDM_LDLGEGL' | 'NGLDM_GLNU' |
| 17 | 'GLSZM_LGZE' | 'Variance' |
| 18 | 'GLSZM_LZLGE' | 'SUVP33' |
| 19 | 'x10thPercentile' | 'SUVP14' |
| 20 | 'Minimum' | 'GLDZM_GLNU' |
| 21 | 'QuartileCoefficientOfDispersion' | 'GLSZM_HGZE' |
| 22 | 'GLCM_InfoMesOfCorr1' | 'NGLDM_DCNU' |
| 23 | 'Median' | 'NGTDM_Busyness' |
| 24 | 'GLSZM_HGZE' | 'GLSZM_LZE' |
| 25 | 'QEnergy' | 'GLDZM_SDLGLE' |
| 26 | 'GLCM_Entropy' | 'InterquartileRange' |
| 27 | 'GLCM_MaximumProbability' | 'Minimum' |
| 28 | ‘GLCM_Energy’ | 'GLDZM_ZDNU' |
| 29 | 'SUVmean' | 'GLCM_DifferenceEntropy' |
| 30 | 'GLSZM_LZE' | 'RobustMeanAbsoluteDeviation' |
| 31 | 'GLCM_SumEntropy' | 'GLSZM_LZHGE' |
| 32 | 'RootMeanSquare' | 'GLDZM_LGDE’ |
| 33 | 'GLDZM_GLV' | 'Range' |
| 34 | 'Variance' | 'GLCM_MaximumProbability' |
| 35 | 'GLCM_Autocorrelation' | 'Kurtosis_hist' |
| 36 | 'NGTDM_Coarseness' | 'Energy' |
| 37 | 'GLCM_Correlation' | 'MedianAbsoluteDeviation' |
| 38 | 'GLRLM_GLN' | 'MeanAbsoluteDeviation' |
| 39 | 'GLCM_Variance' | 'NGTDM_Complexity' |
| 40 | 'NGTDM_Strength' | 'QEnergy' |
| 41 | 'GLDZM_ZDNU' | 'GLDZM_ZDV' |
| 42 | 'GLSZM_LZHGE' | 'GLDZM_LDE' |
| 43 | 'InterquartileRange' | 'GLCM_Entropy' |
| 44 | 'GLCM_InfoMesOfCorr2' | 'NGLDM_HDE' |
| 45 | 'GLDZM_ZP' | 'NGTDM_Contrast' |
| 46 | 'GLRLM_HGRE' | 'GLRLM_LRLGE' |
| 47 | 'GLDZM_GLNU' | 'GLCM_DifferenceVariance' |
| 48 | 'x90thPercentile' | 'x90thPercentile' |
| 49 | 'GLDZM_SDHGLE' | 'GLRLM_LRE' |
| 50 | 'RobustMeanAbsoluteDeviation' | 'GLDZM_SDHGLE' |
| 51 | 'Energy' | 'GLSZM_LZLGE' |
| 52 | 'CoefficientOfVariation' | 'GLDZM_ZDNUN' |
| 53 | 'GLCM_ClusterProminence' | 'GLCM_Dissimilarity' |
| 54 | 'GLSZM_ZP' | 'GLCM_SumEntropy' |
| 55 | 'MedianAbsoluteDeviation' | 'RootMeanSquare' |
| 56 | 'GLRLM_LRHGE' | 'GLDZM_GLV' |
| 57 | 'NGLDM_GLNU' | 'SUVmean' |
| 58 | 'GLRLM_LRE' | 'x10thPercentile' |
| 59 | 'MeanAbsoluteDeviation' | 'NGLDM_HDLGE' |
| 60 | 'GLDZM_ZDE' | ‘Volume’ |
| 61 | 'SUV25' | 'GLSZM_ZP' |
| 62 | 'SUV_N14' | 'GLSZM_LGZE' |
| 63 | 'SUV_N45' | 'GLCM_ClusterProminence' |
| 64 | 'SUV_N90' | 'CoefficientOfVariation' |
| 65 | 'NGLDM_DCNU' | 'GLSZM_ZSV' |
| 66 | 'NGLDM_LDE' | 'Median' |
| 67 | 'GLRLM_LRLGE' | ‘Metabolic tumor volume’ |
| 68 | 'GLCM_Homogeneity1' | 'GLCM_InfoMesOfCorr1' |
| 69 | 'SUVmax' | 'GLDZM_SDE' |
| 70 | 'Range' | 'NGLDM_LDLGEGL' |
| 71 | 'GLCM_Homogeneity2' | 'GLCM_Autocorrelation' |
| 72 | 'GLRLM_RP' | 'NGTDM_Strength' |
| 73 | 'SUVP4' | 'GLDZM_ZDE' |
| 74 | 'NGLDM_HDE' | 'GLCM_Homogeneity1' |
| 75 | 'NGTDM_Busyness' | 'GLRLM_RLN' |
| 76 | 'GLRLM_RLN' | 'GLRLM_SRE' |
| 77 | 'Kurtosis_hist' | 'GLCM_Homogeneity2' |
| 78 | 'SUVP14' | ‘GLCM_Energy’ |
| 79 | 'GLRLM_SRE' | 'QuartileCoefficientOfDispersion' |
| 80 | 'GLDZM_ZDNUN' | 'GLCM_Variance' |
| 81 | 'SUVP33' | 'GLRLM_HGRE' |
| 82 | 'GLDZM_SDE' | 'GLCM_Contrast' |
| 83 | 'GLDZM_ZDV' | 'GLDZM_ZP' |
| 84 | 'GLDZM_LDE' | 'GLSZM_GLV' |
| 85 | ‘Volume’ | 'NGLDM_LDE' |
| 86 | 'NGLDM_HDLGE' | 'GLRLM_LGRE' |
| 87 | 'GLCM_DifferenceEntropy' | 'GLDZM_GLNUN' |
| 88 | 'GLRLM_SRLGE' | 'GLRLM_SRLGE' |
| 89 | 'GLCM_IDN' | 'GLRLM_LRHGE' |
| 90 | 'GLCM_Dissimilarity' | 'GLRLM_RP' |
| 91 | 'GLCM_IDMN' | 'GLSZM_GLN' |
| 92 | 'GLRLM_LGRE' | 'GLCM_IDN' |
| 93 | ‘Metabolic tumor volume’ | 'GLCM_InfoMesOfCorr2' |
| 94 | 'GLCM_DifferenceVariance' | 'GLCM_IDMN' |
| 95 | 'NGTDM_Contrast' | 'NGTDM_Coarseness' |
| 96 | 'NGTDM_Complexity' | 'GLRLM_GLN' |

**Supplemental Table S5***: Accuracy by TNBC Subtyp* ***(%)***

|  | CART | | Naïve Bayes | | SVM | |
| --- | --- | --- | --- | --- | --- | --- |
| Subtype | Baseline | Difference | Baseline | Difference | Baseline | Difference |
| IM | 84.37 | 77.75 | 91.25 | 78.5 | 86.5 | 76.25 |
| BL2 | 72.72 | 70.4 | 83.5 | 73.25 | 75.55 | 71.72 |
| M | 81.25 | 71.25 | 81.25 | 82.5 | 83.75 | 75 |
| BL1 | 87.7 | 68.25 | 96.25 | 85.25 | 91.25 | 82.5 |
| LAR | 62.5 | 75.00 | 72.5 | 82.5 | 65 | 75 |

**Supplemental Table S6:** Performance of ML algorithms

|  |  | Preclinical | | | | Clinical | | | |
| --- | --- | --- | --- | --- | --- | --- | --- | --- | --- |
|  |  | Prediction | | Assessment | | Prediction | | Assessment | |
|  |  | NB | SVM | NB | SVM | NB | SVM | NB | SVM |
| RadSig | F-score | 86.93 | 82.62 | 78.78 | 75.30 | 66.67 | 72.00 | 64.94 | 70.00 |
|  | TPR | 85.81 | 83.33 | 78.00 | 77.38 | 75.00 | 75.00 | 56.82 | 63.64 |
|  | TNR | 86.67 | 78.59 | 78.57 | 72.94 | 53.85 | 69.23 | 80.00 | 80.00 |
|  | PPV | 88.08 | 81.92 | 79.59 | 73.33 | 60.00 | 69.23 | 75.76 | 77.78 |
|  | Accuracy | 86.21 | 81.14 | 78.26 | 75.13 | 64.00 | 72.00 | 67.86 | 71.43 |
| SUV_mean_ | F-score | 53.24 | 62.30 | 58.82 | 76.47 | 51.06 | 61.54 | 50.00 | 56.41 |
|  | Sensitivity (TPR) | 52.00 | 63.33 | 76.92 | 86.67 | 50.00 | 66.67 | 45.45 | 50.00 |
|  | Specificity (TNR) | 53.57 | 57.14 | 31.25 | 57.14 | 57.69 | 53.85 | 60.00 | 70.00 |
|  | Precision (PPV) | 54.55 | 61.29 | 47.62 | 68.42 | 52.17 | 57.14 | 55.56 | 64.71 |
|  | Accuracy | 52.76 | 60.34 | 51.72 | 72.41 | 54.00 | 60.00 | 52.38 | 59.52 |
| SUL_peak_ | F-score | 48.28 | 64.41 | 78.26 | 76.54 | 52.17 | 55.32 | 53.66 | 55.42 |
|  | Sensitivity (TPR) | 50.00 | 65.52 | 84.38 | 83.22 | 50.00 | 54.17 | 50.00 | 52.27 |
|  | Specificity (TNR) | 46.67 | 62.07 | 61.54 | 63.83 | 61.54 | 61.54 | 60.00 | 60.00 |
|  | Precision (PPV) | 46.67 | 63.33 | 72.97 | 70.86 | 54.55 | 56.52 | 57.89 | 58.97 |
|  | Accuracy | 48.28 | 63.79 | 74.14 | 73.79 | 56 | 58 | 54.76 | 55.95 |
| SUV_max_ | F-score | 60.00 | 63.64 | 77.43 | 75.36 | 48.00 | 59.26 | 40.00 | 42.11 |
|  | Sensitivity (TPR) | 60.00 | 63.23 | 86.67 | 86.67 | 50.00 | 66.67 | 36.36 | 36.36 |
|  | Specificity (TNR) | 57.14 | 59.26 | 60.14 | 53.57 | 46.15 | 46.15 | 50.00 | 60.00 |
|  | Precision (PPV) | 60.00 | 64.05 | 69.97 | 66.67 | 46.15 | 53.33 | 44.44 | 50.00 |
|  | Accuracy | 58.62 | 61.38 | 73.86 | 70.69 | 48.00 | 56.00 | 42.86 | 47.62 |

**Supplemental Table S7:** Patient characteristics, pathologic response, and SUV metrics

| **Stage at Diagnosis** | **Grade at Diagnosis** | **pCR** | **BL**  **SUV_mean_** | **BL**  **SUL_P_** | **BL**  **SUV_max_** | **After treatment**  **SUV_mean_** | **After treatment**  **SUL_P_** | **After treatment**  **SUV_max_** | **%Δ**  **SUV_mean_** | **%Δ**  **SUL_P_** | **%Δ**  **SUV_max_** |
| --- | --- | --- | --- | --- | --- | --- | --- | --- | --- | --- | --- |
| IIB (T2N1) | 2 | No | 1.86 | 1.57 | 4.03 | NA | NA | NA | NA | NA | NA |
| IIB (T2N1) | 3 | Yes | 6.59 | 4.58 | 11.27 | 2.57 | 1.46 | 4.75 | -60.97 | -68.11 | -57.82 |
| IIIA (T3N1) | 3 | No | 10.55 | 12.34 | 26.41 | NA | NA | NA | NA | NA | NA |
| IIA (T2N0) | ? | Yes | 8.77 | 7.80 | 21.48 | 2.15 | 1.39 | 2.84 | -75.49 | -82.21 | -86.77 |
| IIB (T2N1) | 3 | No | 3.18 | 1.64 | 5.31 | 3.05 | 1.43 | 4.81 | -4.32 | -13.19 | -9.40 |
| IIB (T2N1) | 3 | No | 2.92 | 1.29 | 4.54 | 1.79 | 0.76 | 2.65 | -38.67 | -41.17 | -41.77 |
|  |  |  | 8.01 | 6.43 | 15.55 | 4.74 | 3.58 | 8.88 | -40.82 | -44.37 | -42.90 |
| IIB (T2N1) | 3 | No | 8.58 | 6.20 | 17.02 | 3.01 | 1.84 | 4.53 | -64.92 | -70.33 | -73.40 |
|  |  |  | 8.91 | 6.62 | 20.11 | 3.32 | 2.11 | 7.70 | -62.72 | -68.17 | -61.72 |
| IIA (T2N0) | 3 | Yes | 2.49 | 1.61 | 5.25 | NA | NA | NA | NA | NA | NA |
| IIA (T2N0) | 3 | Yes | 4.17 | 3.06 | 12.45 | 2.13 | 1.58 | 4.45 | -48.84 | -48.53 | -64.23 |
| IIA (T2N0) | 3 | Yes | 3.89 | 3.12 | 8.62 | 1.64 | 1.25 | 2.27 | -57.76 | -59.91 | -73.72 |
| IIB (T2N1) | 3 | No | 2.85 | 2.59 | 6.28 | 1.67 | 1.15 | 2.77 | -41.45 | -55.68 | -55.97 |
|  |  |  | 2.48 | 2.03 | 5.03 | 1.81 | 1.26 | 2.52 | -27.04 | -37.81 | -49.88 |
| IIA (T2N0) | 3 | No | 8.66 | 7.56 | 21.87 | 2.09 | 1.32 | 4.08 | -75.82 | -82.48 | -81.35 |
| IIB (T2N1) | 3 | Yes | 10.29 | 8.32 | 28.33 | 2.66 | 1.96 | 5.36 | -74.14 | -76.39 | -81.07 |
|  |  |  | 3.64 | 2.02 | 5.65 | 1.49 | 0.87 | 2.53 | -58.93 | -57.11 | -55.17 |
|  |  |  | 8.00 | 5.60 | 16.06 | 2.26 | 1.28 | 3.67 | -71.75 | -77.22 | -77.14 |
| IIA (T2N0) | 3 | Yes | 1.86 | 1.65 | 4.18 | 1.29 | 1.04 | 1.96 | -30.42 | -36.91 | -53.02 |
| IIA (T2N0) | 3 | No | 9.80 | 7.74 | 17.69 | 3.17 | 2.75 | 8.11 | -67.65 | -64.44 | -54.15 |
| IIA (T2N0) | 3 | Yes | 7.19 | 5.21 | 14.57 | 3.86 | 3.00 | 8.27 | -46.36 | -42.49 | -43.23 |
| IIA (T2N0) | 3 | No | 7.98 | 7.26 | 18.46 | 4.31 | 3.32 | 10.57 | -45.99 | -54.32 | -42.74 |
| IIA (T2N0) | 3 | Yes | 2.91 | 2.03 | 5.24 | 1.74 | 1.22 | 2.17 | -40.23 | -40.04 | -58.66 |
| IIB (T2N1) | 3 | Yes | 6.19 | 5.53 | 11.28 | 1.87 | 1.41 | 2.60 | -69.81 | -74.57 | -76.91 |
| IIB (T2N1) | 3 | No | 2.44 | 1.99 | 5.53 | NA | NA | NA | NA | NA | NA |
